# Baicalin Inhibits *Haemophilus Parasuis*-Induced High-Mobility Group Box 1 Release during Inflammation

**DOI:** 10.3390/ijms19051307

**Published:** 2018-04-27

**Authors:** Shulin Fu, Huashan Liu, Xiao Chen, Yinsheng Qiu, Chun Ye, Yu Liu, Zhongyuan Wu, Ling Guo, Yongqing Hou, Chien-An Andy Hu

**Affiliations:** 1 Hubei Key Laboratory of Animal Nutrition and Feed Science, Wuhan Polytechnic University, Wuhan 430023, China; fushulin2016@126.com (S.F.); liuhuashan4526@163.com (H.L.); 3377091@163.com (X.C.); yechun0226@163.com (C.Y.); lyywfy@foxmail.com (Y.L.); zhongywu@163.com (Z.W.); guoling1101@163.com (L.G.); houyq@aliyun.com (Y.H.); AHU@salud.unm.edu (C.-A.A.H.); 2 Hubei Collaborative Innovation Center for Animal Nutrition and Feed Safety, Wuhan 430023, China; 3 Biochemistry and Molecular Biology, University of New Mexico School of Medicine, Albuquerque, NM 87131, USA

**Keywords:** baicalin, *Haemophilus parasuis*, HMGB1, RNA-Seq, inflammation

## Abstract

*Haemophilus parasuis* (*H. parasuis*) can cause Glässer’s disease in pigs. However, the molecular mechanism of the inflammation response induced by *H. parasuis* remains unclear. The high-mobility group box 1 (HMGB1) protein is related to the pathogenesis of various infectious pathogens, but little is known about whether *H. parasuis* can induce the release of HMGB1 in piglet peripheral blood monocytes. Baicalin displays important anti-inflammatory and anti-microbial activities. In the present study, we investigated whether *H. parasuis* can trigger the secretion of HMGB1 in piglet peripheral blood monocytes and the anti-inflammatory effect of baicalin on the production of HMGB1 in peripheral blood monocytes induced by *H. parasuis* during the inflammation response. In addition, host cell responses stimulated by *H. parasuis* were determined with RNA-Seq. The RNA-Seq results showed that *H. parasuis* infection provokes the expression of cytokines and the activation of numerous pathways. In addition, baicalin significantly reduced the release of HMGB1 in peripheral blood monocytes induced by *H. parasuis*. Taken together, our study showed that *H. parasuis* can induce the release of HMGB1 and baicalin can inhibit HMGB1 secretion in an *H. parasuis*-induced peripheral blood monocytes model, which may provide a new strategy for preventing the inflammatory disorders induced by *H. parasuis*.

## 1. Introduction

*Haemophilus parasuis* (*H. parasuis*), the causative agent of Glässer’s disease in pigs, is a Gram-negative bacterium that colonizes the upper respiratory tract of pigs [1]. The disease is characterized by fibrinous polyserositis, polyarthritis and meningitis [2]. *H. parasuis* can cause high morbidity and mortality, resulting in huge economic losses for the pig industry [3]. In recent years, it has become one of the most important bacterial respiratory pathogens, and has received increasing attention from pig producers. So far, 15 serovars of *H. parasuis* have been identified, but up to 20% of isolates cannot be serotyped according to the Kielstein–Rapp–Gabrielson serotyping scheme [4]. Serovars 4, 5 and 13 are the most prevalent serotypes in China [5,6]. In general, *H. parasuis* serovars are considered to be important markers of bacterial virulence [7,8]. Serovar 5 is highly virulent and serovar 4 is considered to be moderately virulent [9]. Because of the large number of *H. parasuis* serovars and uncertainty in the identification of some of them, preventing and controlling infection by *H. parasuis* has become a challenge.

Currently, the pathogenesis of *H. parasuis* infection remains unclear, especially the pathways causing the systemic inflammatory response and vascular injury. However, some virulence-related factors have been demonstrated to play important roles in the pathogenesis of the disease. *H. parasuis* lipooligosaccharides (LOS) can mediate the adhesion of *H. parasuis* to porcine brain microvascular endothelial cells (PBMEC) and are able to induce the release of IL-8 and IL-6 by PBMEC [10]. The contribution of the inner core oligosaccharide of LOS, cytolethal distending toxin (CDT) and the *rfaE* gene are associated with serum resistance and has the ability to adhere to and invade porcine kidney epithelial cells (PK-15) and porcine umbilical vein endothelial cells (PUVEC) [11,12,13]. The *vacJ* gene plays an essential role in maintaining biofilm formation, serum resistance, and adherence to and invasion of PK-15 cells [14]. Deletion of the *arcA* gene resulted in less biofilm mass being produced and reduced *H. parasuis* EP3 strain virulence in mice [15]. Disruption of the *htrA* gene affected resistance to complement-mediated killing and significantly attenuated virulence of *H. parasuis* in the murine model of infection [16]. Despite the numerous virulence-related factors that have currently been discovered, the pathogenesis of inflammation caused by *H. parasuis* still needs to be resolved.

The inflammatory immune response and inflammation injury play important roles in the pathogenesis of Glässer’s disease. Macrophages have important regulatory effects on the inflammatory response [17]. Activation of the inflammation-associated signaling pathway can induce the production of inflammatory cytokines such as IL-6 and IL-8 [18]. High-mobility group box 1 (HMGB1), which is a nuclear protein and is identified as a cytokine, is ubiquitously expressed in many mammalian cells and participates in diverse important intracellular and extracellular functions [19,20]. Previous research has shown that exposure of human bronchial epithelial cells to HMGB1 leads to pro-inflammatory cytokine secretion, enhanced ER-mitochondrial Ca^2+^ transfer and reactive oxygen species (ROS) production [21]. HMGB1 is involved in the pathophysiology of pulmonary fibrosis by causing the release of pro-fibrotic proteins [22]. HMGB1 production is increased in injured mouse spinal cords and can induce neurotoxic inflammation [23]. In addition, lipopolysaccharide (LPS) from *Aggregatibacter actinomycetemcomitans*, *Porphyromonas gingivalis*, and *Escherichia coli* significantly induced HMGB1 secretion from human gingival fibroblasts, which may contribute to periodontal tissue destruction [24]. It has been documented that the HMGB1 inhibitor glycyrrhizic acid can mediate renal injury and inflammatory responses in diabetic rats by regulating the activation of the RAGE/TLR4-related ERK and p38 MAPK/NF-κB signaling pathways [25]. Ethyl pyruvate can suppress acute lung damage through inhibition of NF-κB and HMGB1 following trauma and hemorrhagic shock [26]. Thus, we speculate that HMGB1 may be useful as a valid therapeutic target for controlling *H. parasuis* infection.

Baicalin is the principal component of the flavonoid derivatives in the roots of *Scutellaria baicalensis* Georgi [27]. It has been reported that baicalin has important anti-inflammatory, anti-microbial, and anti-oxidant activities [28]. Baicalin can inhibit biofilm formation, suppress quorum sensing-controlled virulence, and enhance *Pseudomonas aeruginosa* clearance in a mouse peritoneal implant infection model [29]. Baicalin reduced A549 cell injury induced by *Staphylococcus aureus* and protected mice from *S. aureus* pneumonia [30,31]. Baicalin improves the survival of mice with polymicrobial sepsis by suppressing the inflammatory response and lymphocyte apoptosis [32]. Baicalin canprotect CHON-001 cells from IL-1β-induced inflammatory injury through miR-126 downregulation [33]. Baicalin also effectively suppresses the breast cancer metastasis by reversing the epithelial-to-mesenchymal transition [34]. Thus, we speculate that baicalin may be utilized as a novel drug to control the inflammation response or injury evoked by *H. parasuis*.

Our previous research has shown that baicalin can suppress the inflammation response through the NLRP3 inflammasome pathway in LPS-challenged piglet mononuclear phagocytes and the NF-κB and NLRP3 inflammasome pathway in *H. parasuis*-induced piglet mononuclear phagocytes [35,36]. In the present study, we determined the pattern of secretion of HMGB1 in piglet mononuclear phagocytes triggered by LPS and *H. parasuis*. Signaling pathways related to HMGB1 in piglet mononuclear phagocytes infected by *H. parasuis* were also explored by RNA-Seq. In addition, we investigated the effect of baicalin on the secretion of inflammatory cytokines and HMGB1 from piglet mononuclear phagocytes. Our results suggest that baicalin can significantly inhibit the release of HMGB1 in piglet mononuclear phagocytes, which may provide a novel strategy for preventing the inflammation response or injury induced by *H. parasuis*.

## 2. Results

### 2.1. H. Parasuis and Lipopolysaccharide (LPS) Infection-Triggered High-Mobility Group Box 1 (HMGB1) Release in the Piglet Peripheral Blood Monocytes

**Figure 1:**
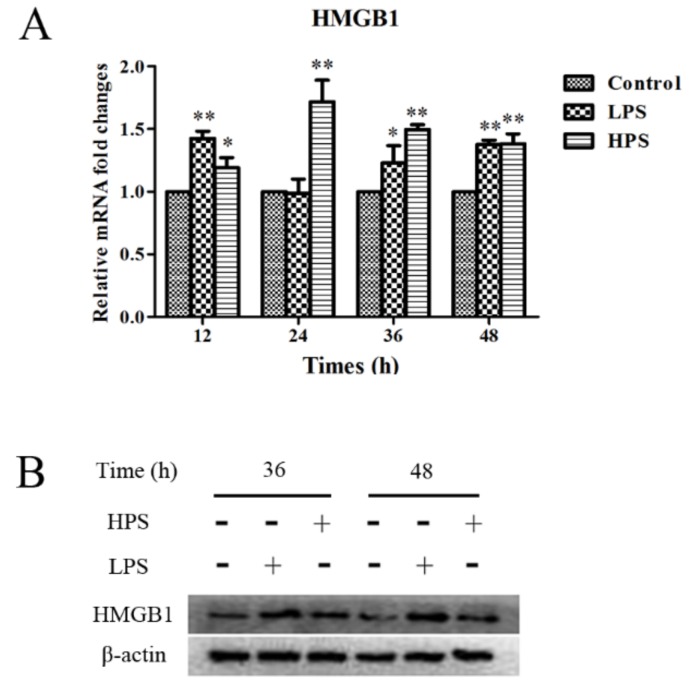
Detection of high-mobility group box 1 (HMGB1) release in the piglet peripheral blood monocytes triggered by *H. parasuis* or lipopolysaccharide (LPS) using the real-time quantitative reverse transcription polymerase chain reaction (qRT-PCR) method (**A**) and Western blot method (**B**). * *p* < 0.05; ** *p* < 0.01; HPS: *H. parasuis*; 36 h: LPS vs. Control (*p* < 0.01) and HPS vs. Control (*p* < 0.01); 48 h: LPS vs. Control (*p* < 0.01) and HPS vs. Control (*p* < 0.05).

To explore the pattern of production of HMGB1 promoted by *H. parasuis* and LPS in detail, the piglet peripheral blood monocytes were infected with *H. parasuis* or LPS for 12 h to 48 h. The results showed that *H. parasuis* could stimulate the production of HMGB1 in the piglet peripheral blood monocytes for 12 h to 48 h compared with the control cells, and the amount of HMGB1 released reached a peak at 24 h before falling at 36 h to 48 h (Figure 1A) (*p* < 0.05). In addition, LPS also could induce HMGB1 secretion at 12, 36, and 48 h (Figure 1A) (*p* < 0.05). Western blot analysis further confirmed the expression of HMGB1 in the cell supernatants when induced at 36 to 48 h (Figure 1B) (*p* < 0.05).

### 2.2. Baicalin Inhibited HMGB1 Release in Piglet Peripheral Blood Monocytes Induced by H. Parasuis

**Figure 2:**
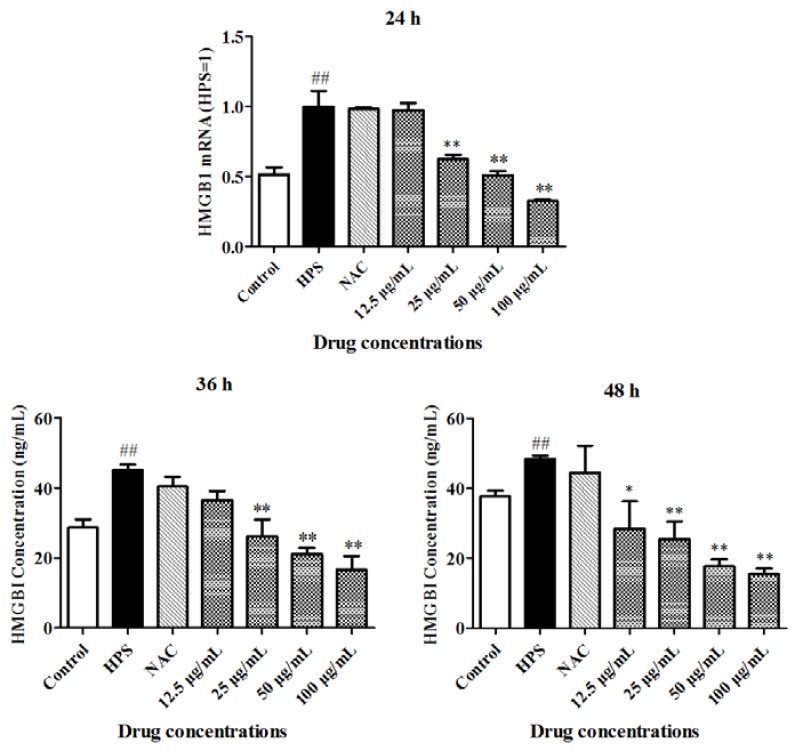
The effect of baicalin on HMGB1 release in piglet peripheral blood monocytes induced by *H. parasuis*. 1 × 10^6^ piglet peripheral blood monocytes were pretreated with baicalin at 12.5, 25, 50, 100 μg/mL or NAC (1 mM/mL) for 1 h. 1 × 10^6^ CFU/mL *H. parasuis* were added to the wells and co-infected for 24, 36 and 48 h. The HMGB1 concentration was measured. ## *p* < 0.01 vs. control. * *p* < 0.05; ** *p* < 0.01; HPS: *H. parasuis*.

After the piglet peripheral blood monocytes were pretreated with 12.5–100 μg/mL baicalin and infected with *H. parasuis* for 24 to 48 h, HMGB1 secretion was measured. These data demonstrated that *H. parasuis* could significantly promote the release of HMGB1 in the piglet peripheral blood monocytes compared with the control cells (Figure 2) (*p* < 0.01). Surprisingly, *N*-acetyl-l-cysteine (NAC) could not trigger the production of HMGB1 compared with the controls (Figure 2). The production of HMGB1 was not significantly attenuated by 12.5 μg/mL baicalin when co-incubated with *H. parasuis* for 24 and 36 h, although it was attenuated after 48 h (Figure 2). In addition, 25–100 μg/mL baicalin could reduce HMGB1 secretion in the piglet peripheral blood monocytes (Figure 2) (*p* < 0.01).

### 2.3. The Effect of LPS on HMGB1 Release in the Piglet Model

**Figure 3:**
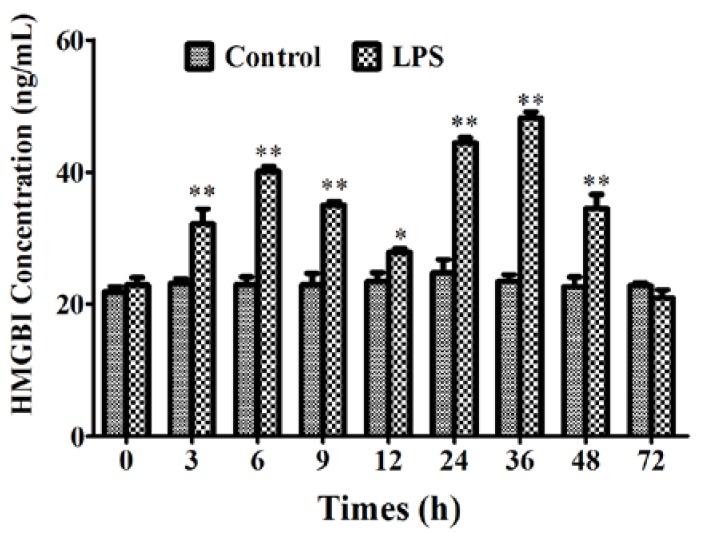
The effect of LPS on HMGB1 release in the piglet model. The piglets were inoculated with LPS for 3, 6, 9, 12, 24, 36, 48, and 72 h, blood samples were collected for the detection of HMGB1 release. * *p* < 0.05; ** *p* < 0.01.

After the piglets were inoculated with LPS for 3, 6, 9, 12, 24, 36, 48, and 72 h, blood samples were collected for the detection of HMGB1 release. The results demonstrated that the levels of HMGB1 secretion significantly increased from 3 to 48 h compared with the control (Figure 3) (*p* < 0.05). HMGB1 release rose to a peak at 36 h and then declined at 72 h (Figure 3).

### 2.4. RNA-Seq Analysis of the Interaction between Host Cells and Bacteria

**Figure 4:**
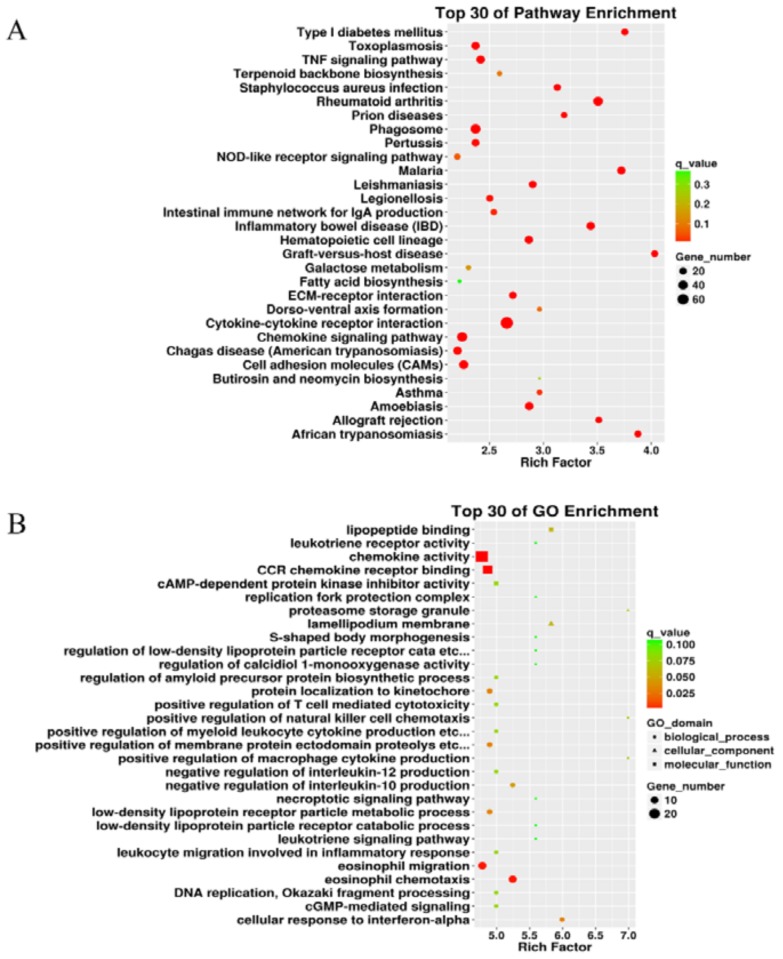
Analysis of the top 30 pathway enrichment (**A**) and gene ontology (GO) enrichment (**B**) by RNA-Seq.

To understand the host-pathogen interaction, we performed RNA-Seq of *H. parasuis*-infected piglet peripheral blood monocytes using the Illumina Hiseq 2000 platform. Then the sequences were aligned against *Sus scrofa* gene sequences (Sscrofa11.1). The results showed that more than 55 million raw reads for every sample were obtained. After data filtering, about 53.7 million reads could be mapped to the reference genome (Table 1), which demonstrated that the high quality of the sequences of the samples obtained could be used for the next analysis. After the cells were infected with *H. parasuis* for 24 h, a total of 982 genes were observed to be significantly altered (fold change ≥ 2, *p* < 0.05), of which 646 genes were up-regulated and 336 genes were down-regulated (Supplemental Table S1). Surprisingly, the HMGB1 gene was up-regulated 0.21-fold. To better explore the host cell response to *H. parasuis*, an enrichment analysis utilizing DAVID was carried out. The gene ontology (GO) enrichment analysis showed that differentially expressed genes involved in the top 30 GO enrichments were related to chemokine activity, CCR chemokine receptor binding, eosinophil migration, and chemotaxis (Figure 4A). The top 30 pathways identified as enriched in the infected cells by the Kyoto Encyclopedia of Genes and Genomes (KEGG) are shown in Figure 4B. The cytokine–cytokine receptor interaction, chemokine signaling pathway, and tumor necrosis factor (TNF) signaling pathway were the most enriched upon host cell infection, which indicated the central importance of the signaling pathways in the pathogenesis of *H. parasuis*.

### 2.5. Analysis of the Association among DEGs of the Main Signaling Pathways Using STRING

**Figure 5:**
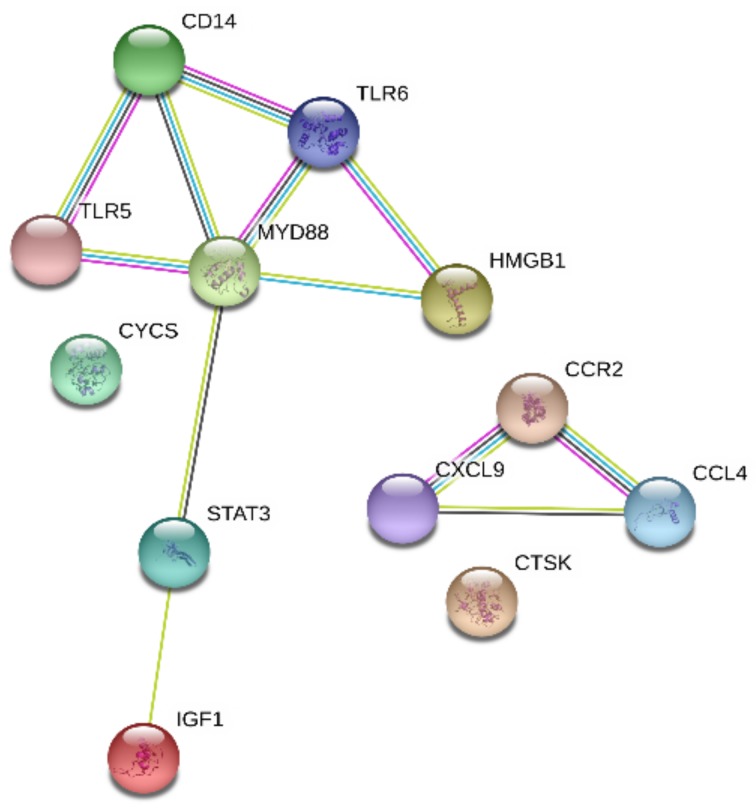
Search Tool for the Retrieval of Interacting Genes/Proteins (STRING) analysis of the relationship between 12 choosed DEGs.

The network of the 12 DEGs which were involved in the main pathways was explored by using the Search Tool for the Retrieval of Interacting Genes/Proteins (STRING) v10 database to show the complex associations between those genes. The analysis demonstrated that most of the DEGs chosed were closely related to each other and showed a coordinated interactive network, but some proteins were not associated with each other (Figure 5). We speculated that the crosstalk of the chosen DEGs triggered inflammation in coordination following *H. parasuis* infection and the network interaction of HMGB1 linked to the possible proteins.

### 2.6. Real-Time Polymerase Chain Reaction (PCR) Verification of DEGs

**Figure 6:**
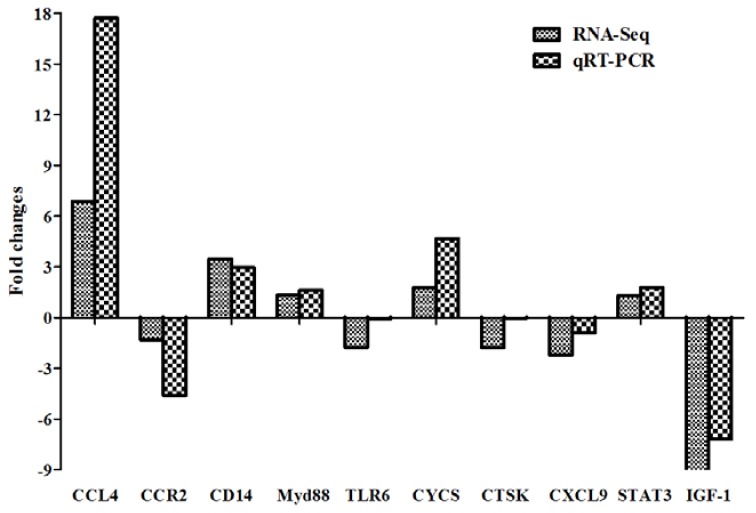
Relative quantification of DEGs for verification by RT-PCR. RT-PCR relative expression levels of selected genes were chosen for the cells infected for 24 h.

Ten genes from the main signaling pathways were choosen for verification of the DEGs data of the RNA-seq by using the real-time quantitative reverse transcription polymerase chain reaction (qRT-PCR) method. The results showed that among the chosen 10 genes, 8 genes (*CYCS*, *CXCL9*, *STAT3*, *IGF-1*, *Myd88*, *CD14*, *CCR2* and *CCL4*) demonstrated similar expression levels compared with the RNA-seq data (Figure 6). And another 2 genes (*CTSK*, *TLR6*) did not display obvious changes in expression levels by using the real-time qRT-PCR method.

## 3. Discussion

Although HMGB1 has recently been reported to be an important immune modulator during bacterial or viral infection [37,38,39], there is so far no evidence that *H. parasuis* can induce HMGB1 release during the infection process of piglet peripheral blood monocytes triggered by *H. parasuis*. In the present study, our work demonstrated that *H. parasuis* can promote the production of HMGB1 in piglet peripheral blood monocytes and, subsequently, may induce inflammatory responses.

Baicalin, a flavonoid, is an important traditional Chinese herb that is extracted from *Scutellaria baicalensis*. Some previous reports have shown that baicalin is an effective treatment forcerebral ischemia [40] and Chikungunya virus infection [41]. Reports also showed that baicalin could attenuate LPS-induced inflammation and apoptosis of cow mammary epithelial cells [42] and LPS-induced injury of intestinal epithelial cells and intercellular tight junctions [43]. However, all of these findings were obtained in vitro. In the present study, we found that HMGB1 release from peripheral blood monocytes was significantly inhibited by 50–100 μg/mL baicalin. Thus, we speculated that one possible effective mechanism provided by baicalin might be related to suppressing HMGB1 release, and then reducing the HMGB1-triggered inflammatory response, but this needs to be investigated in detail. Based on these data, in future we will further explore the effect of baicalin on inflammation responses induced by *H. parasuis* and HMGB1 release triggered by *H. parasuis* in a piglet model.

The innate immune system is the first line of defense and plays an important role in eliminating pathogenic microorganisms [44]. Monocytes are the major innate immune cells that can constitutively express receptors that respond to pathogens [45]. Research has shown that immune cells such as monocytes can release cytokines when induced by pathogenic microorganisms [46]. The over-expression of inflammatory cytokines and their prolonged accumulation can lead to a systemic inflammation response or organ injury [47,48]. HMGB1, a member of the HMG family, is passively secreted from damaged or injured cells following ischemia/reperfusion injury [49], thus it may serve as a damage-associated molecular pattern molecule (DAMP) [50]. It has been documented that pathogen stimulation can result in HMGB1 cytoplasmic translocation, followed by secretion into the extracellular milieu [51]. The over-secretion of HMGB1 extracellularly could lead to severe infections or tissue damage, thereby triggering inflammatory disease [52]. In this study, we used NAC as a positive control. A previous study has shown that NAC could inhibit the translocation of HMGB1 from amnion epithelial cells’ nuclei to cytoplasm [53]. Therefore, we speculated that HMGB1 may be secreted from the nuclei to the extracellular milieu following *H. parasuis* infection, and thus late release of HMGB1 from peripheral blood monocytes may contribute to tissue damage. How HMGB1 production follows *H. parasuis* stimulation and how the secretion of HMGB1 causes damage remain to be determined.

Inflammation plays important roles in the pathogenesis of *H. parasuis* infection. LPS is widely used to construct an inflammation model that can induce the lung and brain to trigger inflammation with the pathological state [54,55]. When monocytes were activated, induced by LPS, it may play as the inflammation central, hence release cytokines [56]. Thus, we chose LPS to induce an inflammation model in piglets. Previous research showed that LPS could induce HMGB1 production in BEAS-2B cells and trigger acute lung injury [57], and that it stimulated HMGB1 secretion in RAW264.7 cells [58]. Consistent with previous research, our results indicated that LPS also could trigger HMGB1 production in the piglet.

In this study, the piglet peripheral blood monocytes were infected with *H. parasuis* for 12 h to 48 h. A long co-incubation may influence the cells’ viability or trigger citotoxicity. We also detected the cell viability at each incubation time and we found that there is no significant citotoxicity induced by *H. parasuis* (data not shown). In this study, the results showed that HMGB1 release by the piglet peripheral blood monocytes induced by LPS at 24 h displayed no significant difference from the control. However, HMGB1 production in piglet blood at 24 h was significantly higher than the control. We speculated that there were other cells existing in the blood that can secrete HMGB1, but which cells provide this function needs to be investigated. Previous reports have shown that HMGB1 is involved in IFN-α production and TNF-related apoptosis-inducing ligand expression by HIV-1-exposed plasmacytoid dendritic cells [59]. HMGB1 can stimulate the production of IL-1, IL-6, and TNF-α in human monocytes [60] and also activate signaling pathways [61,62]. In the present work, our results showed at the RNA level, according to RNA-Seq analysis, that cytokine production, the chemokine signaling pathway, and the TNF signaling pathway were also activated in the peripheral blood monocytes stimulated by *H. parasuis* for 24 h. In addition, HMGB1 expression was up-regulated according to RNA-Seq, although the change fold was very low, which may be related to late cytokine release. However, whether HMGB1 is involved in the promotion of cytokine expression and signaling pathway activation in the piglet peripheral blood monocytes needs confirmation in further research.

Taken together, our study showed that *H. parasuis* can induce HMGB1 release and that baicalin can inhibit HMGB1 secretion in piglet peripheral blood monocytes triggered by *H. parasuis*. These new findings will help to advance our understanding of the molecular mechanisms of *H. parasuis* pathogenesis as well as the anti-inflammatory effect of baicalin. This discovery of baicalin function may provide a new strategy for preventing the inflammatory disorders induced by *H. parasuis*.

## 4. Materials and Methods

### 4.1. Bacterial Strain, Growth Conditions, and Drug

The *H. parasuis* SH0165 isolate used in this study is a highly virulent strain of serovar 5, and was isolated from the lung of a commercial pig with fibrinous polyserositis, arthritis, hemorrhagic pneumonia, and meningitis [5]. The SH0165 isolate was grown in tryptic soy broth (Difco Laboratories, Detroit, MI, USA) supplemented with a final concentration of 10 μg/mL of NAD (Sigma, St Louis, MO, USA) and 10% newborn calf serum (Gibco, Canberra, Australia) at 37 °C.

Baicalin was obtained from the National Institutes for Food and Drug Control (Beijing, China, B110715-201318). When used, baicalin was dissolved in and diluted with RPMI-1640 medium.

### 4.2. Isolation and Culture of Piglet Peripheral Blood Monocytes

The experiments were designed in strict accordance with the recommendations in the China Regulations for the Administration of Affairs Concerning Experimental Animals 1988 and the Hubei Regulations for the Administration of Affairs Concerning Experimental Animals 2005. The protocol was approved by the China Hubei Province Science and Technology Department [permit number SYXK(ER) 2010-0029]. At the end of the study, all experimental piglets were euthanized.

Fifteen 30-day-old, naturally farrowed early-weaned piglets each weighing 6–8 kg (Duroc × Landrace × large white), in which antibodies against *H. parasuis* were negative, were purchased from Wuhan Jinying Livestock Co., Ltd. (Wuhan, China) and used for in vitro and in vivo experiments.

The piglet peripheral blood monocytes were isolated and cultured according to the method our lab previously established [35]. Briefly, piglet heparinized blood from the precaval vein was layered carefully on an equal volume of phosphate-buffered saline (PBS) (pH 7.4) in a conical centrifuge tube, and then an equal volume of mixed blood was carefully layered on the surface of the lymphocyte separation medium (Tian Jin Hao Yang Co,. LTD, Tianjin, China). The suspension was centrifuged at 400× *g* for 20 min at 4 °C. The cells of the lymphocyte layer were collected and washed three times with PBS and centrifuged at 400× *g* for 20 min at 4 °C. Then the cells were resuspended in Roswell Park Memorial Institute (RPMI) 1640 medium (Gibco, Carlsbad, CA, USA) and seeded in a six-well cell culture plate (Costar, Washington, DC, USA). 3 mL of suspension were added to each well, and these were then pre-incubated in a constant temperature incubator at 37 °C with 5% CO_2_ for 3 h in RPMI-1640 containing 10% fetal bovine serum (Gibco, Canberra, Australia). Cells were washed three times with PBS and then washed with pre-warmed RPMI-1640 medium to discard the non-adherent cells. Attached cells (monocytes) were detached using a cell scraper and suspended in RPMI-1640 medium. Mononuclear cells were counted and their viability was determined by trypan blue exclusion.

### 4.3. Western Blot Analysis of the Release of HMGB1

Piglet peripheral blood monocytes (1 × 10^6^) were seeded into 24-well plates and were treated with 1 μg/mL LPS (Sigma, St. Louis, MO, USA), or the plate wells were infected with 1.0 × 10^6^ CFU/mL *H. parasuis*. The MOI was 1:1 according to our previous work [35]. After co-incubation for 24 h and 48 h, the cell supernatants were collected and used to determine the release of HMGB1 using western blot. The cell supernatants were isolated with 12% sodium dodecyl sulfate polyacrylamide gel electrophoresis (SDS-PAGE) and then electrophoretically transferred to a polyvinylidene difluoride (PVDF) membrane. After blocking with 5% skim milk at room temperature for 3 h and washing five times with TBST, the PVDF membrane was incubated with anti-rabbit HMGB1 polyclonal antibody (Abnova, Walnut, CA, USA) or β-actin antibody (Cell Signaling Technology, Danvers, MA, USA) for 12 h at 4 °C. The membrane was washed five times with TBST and incubated with goat anti-rabbit IgG (Cell Signaling Technology, Danvers, MA, USA) at room temperature for 3 h and visualized by utilizing ECL solution (Thermo Pierce ECL, Waltham, MA, USA). The levels of HMGB1 expression and β-actin were measured using a FluorChem FC2 AIC system (Alpha Innotech, San Leandro, CA, USA).

### 4.4. Total RNA Extraction and Real-Time Polymerase Chain Reaction (RT-PCR)

HMGB1 expression at the mRNA level was carried out with the RT-PCR method as previously described with some modifications [63]. Briefly, 1 × 10^7^ piglet peripheral blood monocytes were seeded into 24-well plates and treated with 1 μg/mL LPS or infected with 1 × 10^7^ CFU/mL *H. parasuis* and co-incubated for 12, 24, 36, or 48 h. Then, the cells were collected and total cellular RNA was extracted using TRISOL reagent (Thermo Fisher Scientific, Waltham, MA, USA). Next, the purified RNA was reverse-transcribed to cDNA by utilizing reverse transcriptase (TaKaRa, Beijing, China). The levels of cDNA amplification were measured using a SYBER Green PCR Kit (ABI, Vernon, CA, USA). To explore the effect of baicalin on the release of HMGB1 at the mRNA level, 1 × 10^7^ cells were seeded into 24-well plates and pre-treated with baicalin at concentrations of 12.5, 25, 50, 100 μg/mL or NAC (Sigma, St Louis, MO, USA) 1 mM/mL for 1 h. Then 1 × 10^7^ CFU/mL *H. parasuis* were added to the wells and co-cultured for 24 h. The total RNA was isolated and used for RT-PCR. The primers included β actin (forward, 5′-TGCGGGACATCAAGGAGAAG-3′; reverse, 5′-AGTTGAAGGTGGTCTCGTGG-3′) and HMGB1 (forward, 5′-CTATCCATTGGTGATGTTGC-3′; reverse, 5′-TCCTCCTCTTCCTTCTTTTT-3′). The level of transcription expression of the HMGB1 gene was measured according to the relative quantification of the 2^−ΔΔ*C*t^ method.

### 4.5. RNA-Seq Analysis

To understand the interaction between piglet peripheral blood monocytes and *H. parasuis*, 1 × 10^7^ piglet peripheral blood monocytes were infected with 1 × 10^7^ CFU/mL *H. parasuis* for 24 h. The cellular RNA was extracted for RNA sequence analysis (RNA-Seq) at the Shanghai Biochip Corporation (Shanghai, China). Key pathways and genes were identified by utilizing GO, KEGG and STRING.

### 4.6. Validation by Real-Time Quantitative Reverse Transcription Polymerase Chain Reaction (qRT-PCR)

The cell RNA was extracted and cDNA synthesis was carried out by PrimeScript™ II 1st Strand cDNA Synthesis Kit (TaKaRa, Dalian, China). mRNA expression levels of 10 genes were explored (Table 2). The relative gene expression was measured by using the threshold cycle method. Then, the fold changes were calculated by using the 2^−ΔΔ*C*t^ formula.

### 4.7. Determining the Effect of Baicalin on the Release of HMGB1 Triggered by H. parasuis with Enzyme-Linked Immunosorbent Assay (ELISA)

In brief, 1 × 10^6^ piglet peripheral blood monocytes were seeded into 24-well plates and pretreated with baicalin at 12.5, 25, 50, 100 μg/mL or NAC (1 mM/mL) for 1 h. Then 1 × 10^6^ CFU/mL *H. parasuis* were added to the wells and co-infected for 36 and 48 h. The cell supernatants were collected and the HMGB1 concentration was measured with the HMGB1 enzyme-linked immunosorbent assay (ELISA) kit (Shanghai BlueGene Biotech CO., LTD, Shanghai, China) according to the manufacturer’s protocol.

### 4.8. Detection of the Effect of LPS on the Secretion of HMGB1 in the Piglet Model

To evaluate the effect of LPS on the secretion of HMGB1 in the piglet, three piglets were injected subcutaneously with 2 mL of 500 μg/mL LPS. Then, blood samples were collected at 3, 6, 9, 12, 24, 36, 48 and 72 h. The levels of HMGB1 in the sera were determined with the HMGB1 ELISA kit.

### 4.9. Statistical Analysis

The experimental data are expressed as the mean ± standard deviation (SD). The difference between the two groups was analyzed using the student’s *t*-test. *p* values of <0.05 were considered significant (* *p* < 0.05 and ** *p* < 0.01).

## Figures and Tables

**Table 1:** Statistical summary analysis of RNA-seq datasets of infection cells and control cells.

Samples_ID	All Reads	Mapped Reads	Mapped Pair Reads	Mapped Broken-Pair Reads	Mapped Unique Reads	Mapped Multi Reads	Mapping Ratio
H1	55,854,342	44,980,513	40,035,218	4,945,295	43,121,932	1,858,581	80.53%
H2	54,986,838	44,145,405	39,108,774	5,036,631	42,172,615	1,972,790	80.28%
H3	50,658,012	40,793,438	36,277,250	4,516,188	39,182,384	1,611,054	80.53%
K1	61,627,718	49,131,998	43,393,550	5,738,448	46,888,148	2,243,850	79.72%
K2	55,917,120	44,938,810	39,598,978	5,339,832	42,736,227	2,202,583	80.37%
K3	54,216,750	43,563,887	38,251,636	5,312,251	41,401,511	2,162,376	80.35%

H1, H2, H3: the infected cells; K1, K2, K3: the control cells.

**Table 2:** Primers for qRT-PCR.

Gene	Nucleotide Sequence (5′–3′)	
*β-actin*	Forward	TGCGGGACATCAAGGAGAAG
	Reverse	AGTTGAAGGTGGTCTCGTGG
*CCR2*	Forward	ATGCCCAGTTTTCTACGGGG
	Reverse	CCGGGCACTTGCTTTAGAGA
*CD14*	Forward	CACTGCCTAGTGCCAAGGAT
	Reverse	CCCACGTTCGCTACACTTCT
*Myd88*	Forward	CATCCCTTGGATGTCAGGCA
	Reverse	AAACTGGATATCGCTGGGGC
*TLR6*	Forward	TGTTGACCACAGGGAGGGTA
	Reverse	TGGATCCACATTGCATGGCT
*CYCS*	Forward	CCTCCATGGTCTCTTTGGGC
	Reverse	GGCGGTGGCCAACTTTTACT
*CTSK*	Forward	GCCATTGATGCAAGCCTGAC
	Reverse	ATAGCCTTTGTTGCCCCAGT
*CXCL9*	Forward	AACAGCCCGTGTCAACATGA
	Reverse	GTGGAAAGGTGTGGAATGCG
*STAT3*	Forward	CCCCGTGTCTAATAGGGGAG
	Reverse	ATCCAAGGGGCCAGAAACTG
*IGF-1*	Forward	TGTACTGTGCACCCCTCAAG
	Reverse	AACTCGTGCAGAGCAAAGGAT
*CCL4*	Forward	CTTCACATACACCGTGCGGA
	Reverse	AGACCTGCCTGCCCTTTTTG

## Supplementary Materials

Supplementary materials can be found at http://www.mdpi.com/1422-0067/19/5/1307/s1.
